# Neuropeptide Y Variation Is Associated With Altered Static and Dynamic Functional Connectivity of the Salience Network

**DOI:** 10.3389/fnsys.2021.629488

**Published:** 2021-11-18

**Authors:** Katherine G. Warthen, Robert C. Welsh, Benjamin Sanford, Vincent Koppelmans, Margit Burmeister, Brian J. Mickey

**Affiliations:** ^1^Department of Biomedical Engineering, The University of Utah, Salt Lake City, UT, United States; ^2^Department of Psychiatry, The University of Utah, Salt Lake City, UT, United States; ^3^Department of Psychiatry, University of Michigan, Michigan, MI, United States; ^4^Michigan Neuroscience Institute and Departments of Computational Medicine & Bioinformatics, Human Genetics and Psychiatry, The University of Michigan, Michigan, MI, United States

**Keywords:** neuropeptide Y, dynamic connectivity, LEiDA, graph theory, fMRI

## Abstract

Neuropeptide Y (NPY) is a neurotransmitter that has been implicated in the development of anxiety and mood disorders. Low levels of NPY have been associated with risk for these disorders, and high levels with resilience. Anxiety and depression are associated with altered intrinsic functional connectivity of brain networks, but the effect of NPY on functional connectivity is not known. Here, we test the hypothesis that individual differences in NPY expression affect resting functional connectivity of the default mode and salience networks. We evaluated static connectivity using graph theoretical techniques and dynamic connectivity with Leading Eigenvector Dynamics Analysis (LEiDA). To increase our power of detecting NPY effects, we genotyped 221 individuals and identified 29 healthy subjects at the extremes of genetically predicted NPY expression (12 high, 17 low). Static connectivity analysis revealed that lower levels of NPY were associated with shorter path lengths, higher global efficiency, higher clustering, higher small-worldness, and average higher node strength within the salience network, whereas subjects with high NPY expression displayed higher modularity and node eccentricity within the salience network. Dynamic connectivity analysis showed that the salience network of low-NPY subjects spent more time in a highly coordinated state relative to high-NPY subjects, and the salience network of high-NPY subjects switched between states more frequently. No group differences were found for static or dynamic connectivity of the default mode network. These findings suggest that genetically driven individual differences in NPY expression influence risk of mood and anxiety disorders by altering the intrinsic functional connectivity of the salience network.

## Introduction

Neuropeptide Y (NPY) is among the most abundantly expressed peptides in the brain (Tatemoto et al., 1982; Adrian et al., 1983; Allen et al., 1983), and is known to affect the neural processing of emotion, appetite, and stress (Morgan et al., 2002; Van Den Heuvel et al., 2015; Reichmann and Holzer, 2016). Individuals vary in their expression of NPY, and low levels of NPY have been associated with the development of anxiety (Reichmann and Holzer, 2016), depression (Widdowson et al., 1992; Heilig et al., 2004; Mickey et al., 2011), and posttraumatic stress disorder (PTSD) (Yehuda et al., 2006). We recently showed that healthy individuals with genetically driven low NPY expression exhibit heightened activation of the nucleus accumbens in response to salient stimuli (Warthen et al., 2018). There is also evidence that high NPY levels predispose individuals to attention-deficit hyperactivity disorder (ADHD) (Oades et al., 1998; Lesch et al., 2011; Pjetri et al., 2012). While anxiety, depression, PTSD, and ADHD are associated with changes in the functional connectivity of intrinsic brain networks (Helm et al., 2018), how NPY affects functional connectivity has not been studied.

Functional connectivity techniques allow one to study correlational relationships between brain regions and reveal information about long distance communication in brain networks (van den Heuvel and Hulshoff Pol, 2010). Intrinsic brain networks may be analyzed using graph theory, which is the study of pairwise relationships or sets of relationships (Wang et al., 2010). Traditionally, functional connectivity techniques have assumed that the brain networks are static (Smith, 2012). More recent techniques have been developed to reveal dynamic connectivity, giving valuable information about how correlational relationships between brain regions change over time (Hutchison et al., 2013; Lurie et al., 2020). In this paper we investigate how NPY expression effects both the static and dynamic connectivity of the brain using graph theoretical methods and Leading Eigenvector Dynamics Analysis (LEiDA) (Cabral et al., 2017), respectively.

We focus here on connectivity within the salience network and within the default mode network, both of which have been implicated in anxiety and depression (Kaiser et al., 2015b), as well as ADHD (Norman et al., 2017). Depression has been associated with unstable connectivity (Wise et al., 2017) and hyperconnectivity (Liston et al., 2014; Kaiser et al., 2015a) in the default mode network. Anxiety has been associated with both lower (Geng et al., 2016; Xu et al., 2019) and higher intra-salience network functional connectivity (Sylvester et al., 2012), and an increase in salience network connectivity has been reported in children with greater behavioral inhibition (Taber-Thomas et al., 2016). ADHD has presented with altered connectivity to important nodes in the salience and default mode network (Fair et al., 2013) as well as increased temporal variability in default mode connectivity (Mowinckel et al., 2017).

Here we investigate differences in resting functional connectivity between two extreme groups of healthy young adults who were selected by NPY genotype (Warthen et al., 2018). Subjects in the high-NPY and low-NPY groups are genetically predisposed to express high and low levels of NPY, respectively. We use multiple techniques to evaluate functional connectivity within the salience network and default mode network. First, we used graph theory to evaluate static resting functional connectivity within the salience network and default mode network. Second, a dynamic connectivity technique [LEiDA, (Cabral et al., 2017)] was used to determine group differences in dynamic network patterns within the salience network and default mode network. Finally, we performed an exploratory seed-based analysis of connectivity strength of the nucleus accumbens (NAc) with the whole brain, based on previously discovered group differences in activation of this region during a monetary incentive delay task (Warthen et al., 2018). We hypothesized that low-NPY subjects would have a more tightly knit salience network due to their previously reported stronger NAc responses to salient stimuli (Warthen et al., 2018). Although increased NAc activity has not yet been tied to a more functionally connected salience network, early functional connectivity studies have indicated that higher activity in certain regions has been related to higher functional connectivity within their primary networks, possibly due to the plastic nature of the functional networks and their components (Greicius et al., 2003). We also hypothesized that high-NPY subjects would display faster switching rates between states, or defined connectivity patterns, within the salience network and the default mode network because of evidence that high levels of NPY predispose to hyperactivity or ADHD (Lesch et al., 2011; Warthen et al., 2018). Although intra-network variability in the salience network in hyperactivity or ADHD is not well characterized, inter-network variability has been shown to be increased in subjects with ADHD possibly indicating more volatile functional connectivity overall (Cai et al., 2018; Wang et al., 2018).

## Materials and Methods

### Participants and Design

Healthy adults (*n* = 222), aged 18–22 years, were genotyped and those with pre-specified NPY genotypes participated in an imaging visit (*n* = 53). This visit included questionnaires, drug and pregnancy screens, and task and resting state functional magnetic resonance imaging (fMRI). The characteristics of the subject sample and results of the task fMRI have been reported previously (Warthen et al., 2018).

### Subject Selection by Genotyping

Subjects were healthy as determined by the Mini International Neuropsychiatric Interview, drug screen, and pregnancy screen. The genotyped groups were the same as we described previously (Warthen et al., 2018). In brief, 6 polymorphic markers were determined by PCR followed by Sanger sequencing (Sanger et al., 1977) to classify subjects with unambiguously low haplotypes, unambiguously high NPY haplotypes, or intermediate, rare or ambiguous combinations of haplotypes. Thirty-one low-NPY and 22 high-NPY subjects underwent MRI.

### Image Acquisition and Preprocessing

Blood oxygenation-level-dependent (BOLD) responses were measured with T2^∗^ weighted imaging (TR = 2 s, TE = 28 ms, flip angle = 90°, 39 transverse slices, slice thickness = 3.5 mm, slice gap = 0 mm, FOV = 64 × 64 matrix, 3.75 × 3.75 mm in-plane resolution) on a 3.0-T Philips Ingenia scanner (Best, Netherlands) with a 15-channel head coil using single-shot echo-planar imaging. Participants were given a focus point and asked to relax with eyes open during a resting state acquisition, during which 300 volumes were collected over 10 min. Resting state acquisition for both groups took place after the monetary incentive delay task described in Warthen et al. (2018). The first 5 volumes of each session were discarded to allow for image stabilization. Slice-time correction was applied with SPM12 (v7219) using the middle slice as the reference. A rigid-body least-squares two-pass procedure was used for motion correction that first registered images to the first image of the first run, and then to the mean image that was calculated after the first pass. Images were interpolated with a 4th degree B-spline. A high-resolution T1-weighted image (turbo-field-echo, TR = 9.8 ms, TE = 4.6 ms, flip angle = 8°, 1 × 1 × 1 mm voxel size) was co-registered to the mean functional image with a rigid-body transform using a normalized mutual-information cost function. The registered T1 was segmented with unified segmentation (Ashburner and Friston, 2005) in SPM12 (v7219). The image was segmented into gray matter, white matter, and cerebrospinal fluid. Gray and white matter images were normalized to MNI space with DARTEL using the provided MNI-space template in the VBM8 toolbox. The estimated warp was then applied to the motion-corrected functional images, which were then resliced to 3 × 3 × 3 mm voxels. Smoothing was performed with an isotropic kernel (5 mm full width half maximum). Twenty-four subjects with excessive head motion (mean frame displacement > 0.20 mm) were excluded from analysis, leaving 29 subjects available for final analyses.

### Connectivity Preprocessing

Resting-state time-series data were preprocessed with a custom toolbox in MATLAB (ConnTool, authored by Dr. Welsh, available upon request) (Jelsone-Swain et al., 2010; Jacobs et al., 2014). Time-series data were quadratically detrended, and regressed against six realignment parameters from motion correction along with their first derivatives and quadratic terms. Additionally, physiological artifacts were minimized through incorporation of COMPCOR by regressing out the signals from cerebral spinal fluid and white matter (Behzadi et al., 2007). Bandpass filtering was performed with a fast Fourier transform filter between 0.01 and 0.10 Hz. Preprocessing steps followed guidelines put forward by Lindquist et al. (2019). Pearson correlation coefficients were calculated between the average BOLD signal in the NAc ROI and all other voxels in the brain. To account for varying degrees-of-freedom in resting-state time-series data due to autocorrelative structures, we incorporated a recently developed technique (Afyouni et al., 2019) into ConnTool to produce the final z-score images. This technique corrects for inflated correlation coefficients due to autocorrelation as well as instantaneous and lagged cross-correlation (known as “xDF” z-scores). Each individual xDF corrected z-score image was included in a second-level SPM12 random effects analysis, to test for whole-brain group differences in connectivity strength in the NAc and dACC maps.

### Seed-Based Functional Connectivity

Region of interest “seed-based” connectivity was evaluated for the nucleus accumbens (NAc) and dorsal anterior cingulate cortex (dACC) because of previously observed group differences (low-NPY vs. high-NPY) in the BOLD signal in the NAc during a monetary incentive delay task (Warthen et al., 2018) and previous associations of dACC connectivity with risk for depression (Wagner et al., 2017; Schwartz et al., 2019). The NAc region of interest was created from a standard atlas [wfu pickatlas, (Maldjian et al., 2003)] and dilated by 2 voxels to allow for shift in activation peaks (Sacchet and Knutson, 2013; Warthen et al., 2018), thresholded at 0.5 and then binarized. The dACC region of interest was created from the Desikan atlas (Desikan et al., 2006) and also thresholded at 0.5 and then binarized.

### Network Definition

**FIGURE 1:**
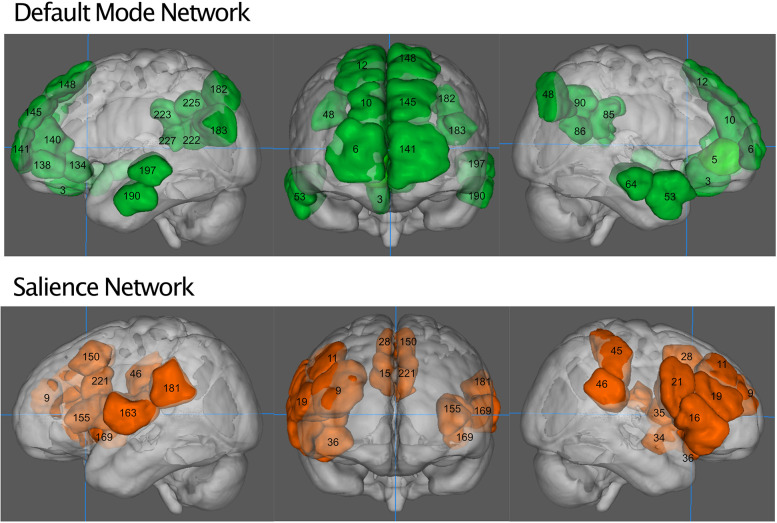
The default mode network (green, **top**) and salience network (orange, **bottom**). Numbers indicate regions of the Shen atlas.

We defined salience network mask similarly to Vinod Menon (2015), from a publicly available functional connectivity map based on 1,000 healthy subjects (Yeo et al., 2011), with a 6 mm spherical seed at (36, 18, 4 mm) and its homolog (−36, 18, 4 mm), obtained from neurosynth.org. The salience network was thresholded to above 0.3 (Pearson’s r) and then binarized. We parcellated the resting state fMRI data from our subjects into 268 nodes as defined by Shen et al. (2013). Nodes were considered part of the salience network if they overlapped the neurosynth generated mask by at least 20% of their volume. This resulted in the inclusion of 20 nodes (Table 1). The default mode network was defined similarly, from a connectivity map from neurosynth.org with a seed at (4, −54, 28 mm) in the posterior cingulate cortex, as by Uddin et al. (2009). Nodes that overlapped by 20% were included, resulting in a 26-node network (Table 1). The objective way that we identified the networks did result in some asymmetry between hemispheres, we have chosen to leave this asymmetry in our network analyses so as not to bias the findings and remain true to the 20% overlap rule. Figure 1 shows the anatomical locations of these regions in Montreal Neurological Institute space. Here we focus only on the salience network and default mode network rather than all 268 nodes in the atlas because of a previously demonstrated effect of NPY on nodes of the salience network (Brown et al., 2000; Adewale et al., 2007; Quarta et al., 2011) in animal studies, as well as the relationship of network patterns in the default mode network and depression and anxiety (Sylvester et al., 2012; Qiao et al., 2017).

**TABLE 1:** Shen atlas regions of interest.

**Salience network**	**Center of mass**	**Default mode network**	**Center of mass**
9	(29, 51, 19)	3	(5, 35, −18)
11	(38, 35, 31)	5	(8, 46, −2)
15	(7, 21, 31)	6	(15, 65, 4)
16	(54, 25, 1)	10	(9, 53, 24)
19	(48, 36, 15)	12	(15, 37, 49)
20	(37, 21, 6)	48	(48, −62, 35)
21	(55, 10, 22)	53	(53, 11, −22)
28	(6, 14, 49)	64	(56, −8, −14)
34	(42, 5, −8)	85	(5, −39, 27)
35	(41, 4, 7)	86	(12, −57, 18)
36	(38, 21, −10)	90	(6, −57, 38)
45	(53, −27, 41)	134	(−5, 29, −10)
46	(58, −29, 20)	138	(−7, 48, −6)
150	(−5, 18, 46)	140	(−6, 48, 12)
155	(−32, 22, 6)	141	(−12, 65, 4)
163	(−57, −3, 7)	145	(−10, 56, 30)
168	(−39, 2, 10)	148	(−11, 34, 52)
169	(−39, 8, −5)	176	(−9, −71, 32)
181	(−59, −26, 22)	182	(−42, −66, 42)
221	(−5, 13, 29)	183	(−51, −56, 20)
		190	(−58, −6, −23)
		197	(−57, −15, −7)
		222	(−8, −59, 18)
		223	(−5, −36, 32)
		225	(−6, −54, 37)
		227	(−7, −42, 13)

### Static Connectivity Analysis

We used the BRAPH [BRain Analysis using graPH theory (1.0.0) (Mijalkov et al., 2017)] package for Matlab (R2015b) to calculate measures of network integration (characteristic path length, global efficiency), network segregation (clustering coefficient, modularity), and small-worldness (Rubinov and Sporns, 2010; Sporns, 2011). These measures allow us to compare the high and low-NPY groups in terms of global network cohesion and local communication of the network, as both may be important in functional differences. *Characteristic path length* is the average distance between a node and all other nodes in terms of edge length traversed. *Efficiency* is related to characteristic path length, but is the inverse of average shortest path length. The *clustering coefficient* is the fraction closed triangles to open triangles around an individual node; where triangles occur when three nodes are all connected to each other by edges. Clustering coefficient therefore gives an idea of how much grouping, or local coherent oscillation, is occurring around a given node. *Modularity* is used to measure how a network is divided into subunits, or communities, by comparing the number of edges inside a cluster with the number of edges that would be found in that cluster in a random graph. *Small-worldness* measures the balance between local grouping and global cooperation in a network. It consists of the ratio of the normalized clustering coefficient over the normalized characteristic path length. These measures (portrayed in Supplementary Figure 1) characterize how closely certain regions of interest are connected. We also investigated node-specific measures of strength (sum of all edge weights feeding into a node) as well as eccentricity (maximum length from one node to its farthest connection) to estimate how our nodes (brain regions) are connected in a local and global network sense. We used a weighted undirected graph with Pearson correlation, and a correlation threshold of zero.

### Dynamic Connectivity Analysis

We were primarily interested in within-network state representation and switching activity within the salience and default mode networks, so we employed the Leading Eigenvector Dynamics Analysis (LEiDA) method^1^ described by Cabral et al. (2017) and Deco et al. (2019). LEiDA is a data-informed way of defining brain states and switching rates between brain states based on identification of large-scale functional connectivity patterns. A leading eigenvector is a vector containing information describing the largest variations in a matrix and is used to find the dominant connectivity pattern at a certain timepoint in vector form. The dominant connectivity pattern can then be reconstructed with the outer product of the vector, where each element of the vector is multiplied by every other element of the vector forming a matrix.

Dynamic connectivity patterns were determined as by Cabral et al. (2017). Leading eigenvectors were calculated at each fMRI time point for each person from the BOLD Phase Coherence Connectivity (Glerean et al., 2012), and the leading eigenvectors combined for all subjects were used for state calculation in each network. Repeating functional connectivity states were determined with k-means clustering (Lloyd, 1982), with a Dunn’s index (Dunn, 1974) determining the optimal number of states.

### Statistical Analyses

Statistical comparisons of network measures were performed in R (version 3.6.1). Static and dynamic network measures were calculated per individual and group differences were determined with a standard two-sided *t*-test. Static and dynamic measures for both networks were normally distributed as determined by a Shapiro-Wilk test. Effect sizes were calculated with Cohen’s d (“cohen.d” function, “effsize” package, version 0.7.6). For tests of the primary hypothesis of NPY group differences in connectivity, Holm’s method was used for multiple-comparison correction (“p.adjust” function, Base R).

Several additional exploratory analyses were performed. Associations between static and dynamic network measures in the salience network as well as associations between traits (Table 2) and network measures were tested with a linear model (“lm” function, “lme4” package, version 1.1.21 in R) while controlling for the effects of NPY group. Sex differences were tested with a standard two-sample *t*-test. Salience condition BOLD contrasts (high-NPY vs. low-NPY) from a previously published monetary incentive delay task (Warthen et al., 2018) for the nucleus accumbens, dorsal anterior insula, and dorsal anterior cingulate cortex were also tested for associations with static and dynamic network measures with a linear model while controlling for NPY group because of the prominence of the nucleus accumbens, anterior insula, and dorsal anterior cingulate cortex in the salience network (Menon, 2015).

**TABLE 2:** Demographic, physiological, and clinical characteristics of the sample.

	**All (*n* = 29)**		**Low NPY (*n* = 17)**		**High NPY (*n* = 12)**	
	**Mean or n**	**SD or%**	**Mean or n**	**SD or%**	**Mean or n**	**SD**
Age	20.31	1.24	20.53	1.23	20.00	1.24
Sex, n female (%)	14	48.3%	8	47.1%	6	50.0%
**Race**						
White, n (%)	23	79.3%	14	82.4%	9	75.0%
Asian, n (%)	5	17.2%	2	11.8%	3	25.0%
Black, n (%)	1	3.4%	1	5.9%	0	0.0%
**Physiological measures**						
Heart rate (per minute)	70.41	11.38	70.71	12.01	70.00	10.92
Systolic BP (mmHg)	114.28	14.23	116.94	13.69	110.50	14.71
Diastolic BP (mmHg)	64.48	10.08	66.18	9.41	62.08	10.92
Respiratory rate (per minute)	16.86	1.25	16.53	1.12	17.33	1.30
Height (cm)	168.62	8.61	168.77	9.16	168.40	8.15
Weight (kg)	66.12	13.80	65.11	11.84	67.54	16.64
Body mass index (kg/m2)	23.13	3.43	22.78	3.08	23.61	3.97
**Trait measures**						
NEO-PI-R neuroticism	83.62	29.78	87.23	27.16	78.50	33.71
NEO-PI-R extraversion	116.45	21.38	117.82	22.94	114.50	19.76
NEO-PI-R openness	116.83	20.94	115.88	22.76	118.17	18.94
NEO-PI-R agreeableness	119.45	19.20	115.94	20.64	124.42	18.38
NEO-PI-R conscientiousness	120.21	20.41	115.71	20.64	126.58	19.12
BIS-BAS behavioral inhibition	19.04	4.36	19.18	5.13	18.78	2.59
BIS-BAS reward responsiveness	17.58	1.70	17.53	1.78	17.67	1.66
BIS-BAS drive	11.15	2.09	11.00	2.45	11.44	1.24
BIS-BAS fun seeking	11.81	2.21	11.65	2.18	12.11	2.37
Appetitive motivation scale	14.42	2.37	14.24	2.28	14.78	2.64
SPSRQ reward	11.92	3.70	12.59	3.88	10.67	3.16
SPSRQ punishment	10.42	5.50	10.59	6.18	10.11	4.26
**State measures**						
PANAS positive^*a*^	30.36	6.14	29.76	6.18	31.27	6.26
PANAS negative^*a*^	12.14	2.45	12.00	2.37	12.36	2.66
PHQ-9^*a*^	2.79	2.04	2.88	2.32	2.64	1.63
CESD^*a*^	6.75	4.61	6.76	3.60	6.73	6.05
Perceived Stress Scale^*a*^	10.79	5.88	10.71	6.15	10.91	5.72
Beck Anxiety Inventory^*a*^	6.81	6.93	6.76	6.57	6.90	7.88

*NEO-PI-R, Neuroticism, Extraversion, Openness Personality Inventory—Revised; BIS-BAS, Behavioral Inhibition and Approach Scales; SPSRQ, Sensitivity to Punishment and Sensitivity to Reward Questionnaire; PANAS, Positive and Negative Affect Schedule; PHQ-9, Patient Health Questionnaire; CESD, Center for Epidemiologic Studies Depression Scale; No significant differences between High and Low NPY as determined by a Mann-Whitney test.*

*^*a*^Missing data for 1–2 High NPY subjects.*

## Results

### Subjects

Twenty-nine subjects were genotyped as high-NPY or low-NPY and provided resting functional MRI data that survived quality-control procedures. The final groups of included subjects did not vary in average frame displacement (*p* > 0.05). No group differences were found for any demographics, physiological, trait, or state measures (Table 2).

### Static Connectivity: Salience Network

**FIGURE 2:**
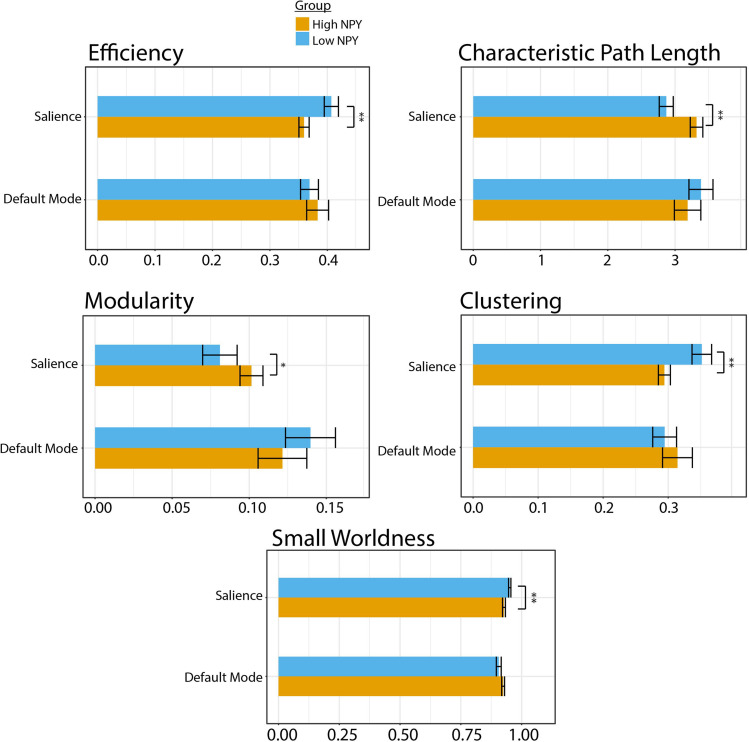
Graph theory parameters for high and low NPY groups in the salience and default mode network. Error bars represent standard error of the mean. ^∗^*p <* 0.05, ^∗∗^*p <* 0.001 as calculated by a two-sided *t*-test.

Low-NPY subjects showed higher values of salience network integration, through shorter characteristic path length (*p* = 0.0012 (uncorrected), *d* = 1.27) and higher global network efficiency [*p* = 0.0065 (uncorrected), *d* = 1.063; Figure 2]. High and low-NPY groups showed split results in measures of network segregation, with high-NPY subjects displaying higher modularity [*p* = 0.041 (uncorrected), *d* = 0.76], but low-NPY subjects having higher clustering [*p* = 0.0025 (uncorrected), *d* = 1.16]. Low-NPY subjects were found to have higher measures of small worldness [*p* = 0.0030 (uncorrected), *d* = 1.17] (Figure 2). These effects all survived Holm’s correction for multiple comparison (characteristic path length, *p* = 0.006; global efficiency, *p* = 0.013; modularity, *p* = 0.041; clustering, *p* = 0.0010; small worldness, *p* = 0.010).

**FIGURE 3:**
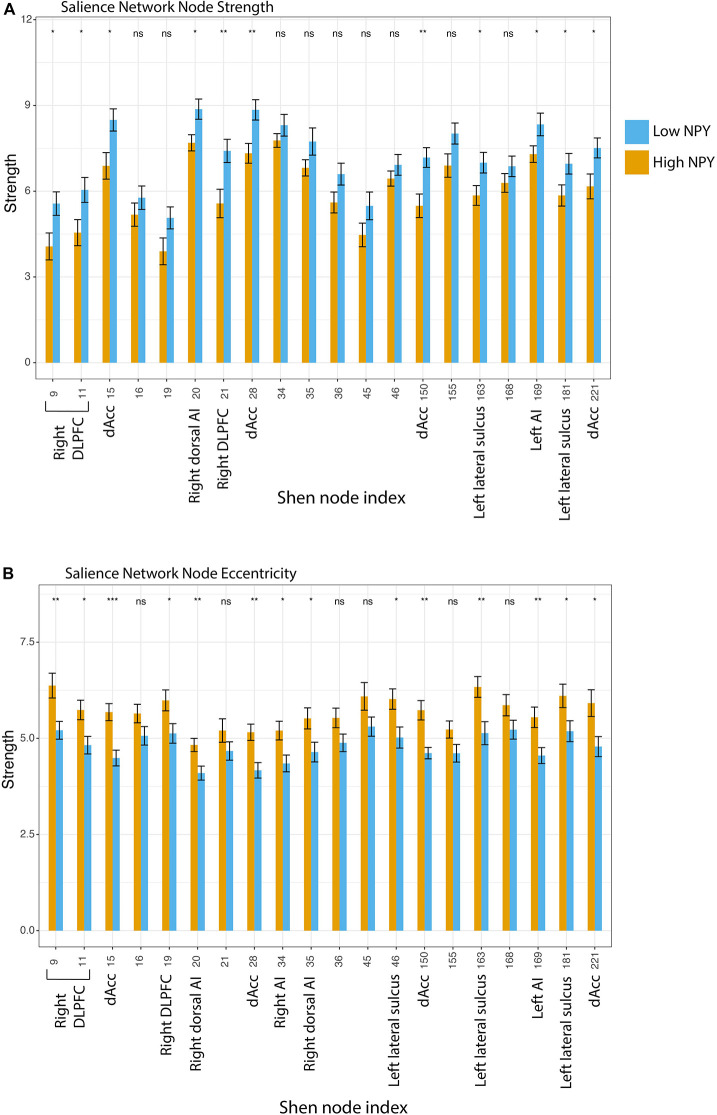
**(A)** Salience network node strength, where Low NPY > High NPY. **(B)** Salience network node eccentricity, where High NPY > Low nPY. *p < 0.05, **p < 0.001 as calculated by a two-sided *t*-test. Regions: 9, Right DLPFC; 11, Right DLPFC; 15, Right dACC; 34, Right Al; 35, Right Dorsal Al; 36, Right VLPFC; 45, Inf. Parietal Lobule; 46, Left lateral sulcus; 150, Left dACC; 155, Left Dosal Al; 163, Left lateral sulcus; 168, Left Al; 181, Left Lateral sulcus; 221, Left dACC.

To further evaluate the underlying causes of these group differences, we tested graph-theoretic metrics at the level of individual nodes (eccentricity and strength). Although not surviving Holm’s correction for multiple comparison for twenty regions, higher node strength was found in the low-NPY group in the bilateral dACC (region 15: *p* = 0.24, 28: *p* = 0.094, 150: *p* = 0.088, 221: *p* = 0.36 after Holm’s correction for multiple comparisons), the right dorsolateral prefrontal cortex (region 9: *p* = 0.36, 11: *p* = 0.026, 21: *p* = 0.16), the right dorsal anterior insula (region 20: *p* = 0.24), the left anterior insula (region 169: *p* = 0.45), and the left lateral sulci (region 163: *p* = 0.36, 181: *p* = 0.45) (Figure 3). Higher eccentricity was found in the high-NPY group in the bilateral dACC (region 15: *p* = 0.011, 28: *p* = 0.041, 150: *p* = 0.024, 221: *p* = 0.17 after Holm’s correction for multiple comparisons), the right dorsolateral prefrontal cortex (region 9: *p* = 0.11, 11: *p* = 0.16, 19: *p* = 0.25), the right anterior (region 34: *p* = 0.17) and dorsal anterior insula (region 35: *p* = 0.25), the left anterior insula (region 169: *p* = 0.11), the left lateral sulci (region 46: *p* = 0.17, 163: *p* = 0.10, 181: *p* = 0.25) (not significant after correction) (Figure 3). Additionally average local efficiency across all nodes within the salience network was found to be significantly different (*p* < 2 × 10 ^–16^). Further details can be found in Supplementary Material.

### Static Connectivity: Default Mode Network

No significant differences were found between high-NPY and low-NPY groups in network integration [global efficiency *p* = 0.43 (uncorrected), *d* = 0.32; characteristic path length *p* = 0.42 (uncorrected), *d* = 0.32], network segregation [clustering *p* = 0.41 (uncorrected), *d* = 0.32; modularity *p* = 0.28 (uncorrected), *d* = 0.42], or small-worldness (small worldness *p* = 0.21 (uncorrected), *d* = 0.45) in the default mode network (Figure 2). However, average local efficiency across all nodes within the default mode network was found to be significantly different (*p* = 0.026).

### Dynamic Connectivity: Salience Network

**FIGURE 4:**
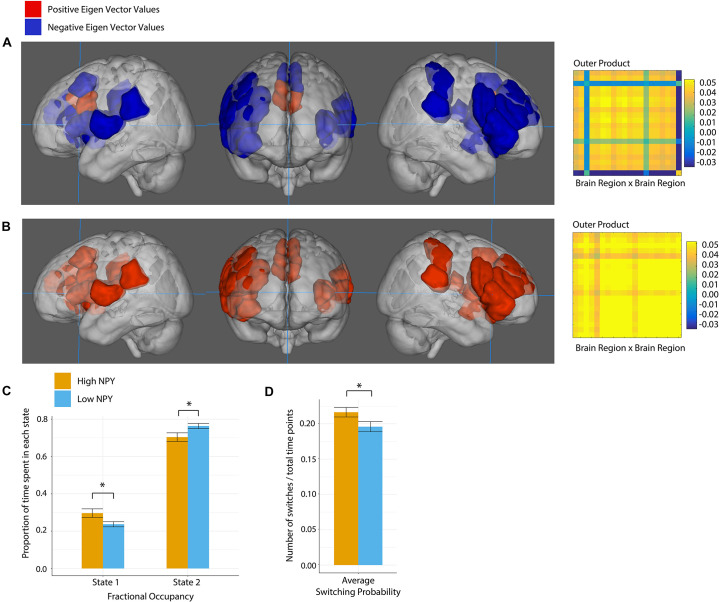
Salience network state 1 **(A)**, and state 2 **(B)**, where anatomical regions are colored by eigen vector value. Matrices show the outer product of the state defining eigen vector. **(C)** Fractional occupancy of high and low NPY groups in state 1 and state 2 of the salience network. **(D)** Switching probability between network states. Error bars represent standard error of the mean. *p < 0.05 as calculated by a two-sided *t*-test.

The salience network was best described by two states according to k-clustering and Dunn’s index. Figure 4 shows each state along with its connectivity matrix representation. Positive and negative eigenvector values represent functional grouping of nodes within the state.

In state 1, the regions centered on dACC were more out of phase with the rest of the nodes in the salience network. In State 2, all nodes in the network were coherent.

High-NPY subjects spent more time in state 1 than low-NPY subjects, conversely low-NPY subjects spent more time in state 2 than high-NPY subjects [*p* = 0.038 (uncorrected), *d* = 0.89]. High-NPY subjects also displayed a higher switching probability between the two states [*p* = 0.047 (uncorrected), *d* = 0.75].

### Dynamic Connectivity: Default Mode Network

**FIGURE 5:**
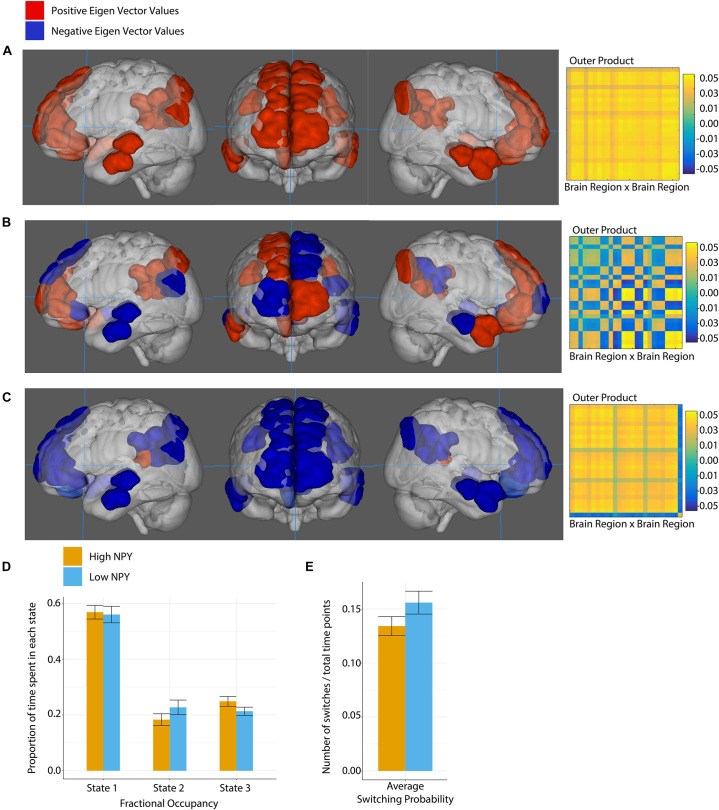
Default mode network state 1 **(A)**, state 2 **(B)**, and state 3 **(C)**, where anatomical regions are colored by eigen vector value. Matrices show the outer product of the state defining eigen vector. **(D)** Fractional occupancy of high and low NPY groups in state 1, state 2, and state 3 of the default mode network. **(E)** Switching probability between network states. Error bars represent standard error of the mean. No significant effects as measured by a two-sided *t*-test.

The default mode network was best described by 3 states, shown in Figure 5. In DMN state 1, all the regions of interest were closely correlated. In state 2, we see two distinct groupings. The first set of regions that includes the right anterior frontal cortex, the left dorsal anterior frontal cortex, left anterior and posterior temporal lobe as well as the right posterior temporal lobe, the right posterior cingulate cortex, and the left temporal parietal junction. The second set of regions includes the left anterior frontal cortex, the right dorsal anterior frontal cortex, the right anterior temporal lobe, the left posterior cingulate cortex and the left and right inferior parietal lobes. In the third state, part of the posterior cingulate cortex (−7, −42, 13) (region 227) is anti-correlated with the rest of the network.

No significant differences between the high-NPY and low-NPY groups were found for fractional occupancy in any of the states, or the average switching probability (*p* > 0.05, two-sided *t*-test). Both groups spent a majority of time in state 1. Switching probabilities between each state in the DMN were tested for group differences; none were found that survived correction for multiple comparison. Details can be found in Supplementary Material.

### Static Connectivity Association With Dynamic Connectivity in the Salience Network

Salience network switching probability was negatively associated with global efficiency [*p* = 6.1 × 10^–5^ (uncorrected), Pearson’s *r* = −0.75] and clustering [*p* = 5.4 × 10^–4^ (uncorrected), Pearson’s *r* = 0.68], but positively associated with characteristic path length [*p* = 2.1 × 10^–4^ (uncorrected), Pearson’s *r* = 0.72] while controlling for NPY group. Fractional occupancy was also negatively associated with global efficiency [*p* = 2.6 × 10^–4^ (uncorrected), Pearson’s *r* = 0.70] and clustering [*p* = 0.0017 (uncorrected), Pearson’s r = 0.63], but positively associated with characteristic path length [*p* = 9.3 × 10^–4^ (uncorrected), Pearson’s *r* = 0.66] and modularity [*p* = 0.030 (uncorrected), Pearson’s *r* = 0.37] while controlling for NPY group.

### Functional Connectivity of the Nucleus Accumbens and Dorsal Anterior Cingulate Cortex

We compared seed-based functional connectivity from the NAc and dACC between groups in an exploratory analysis (Supplementary Figure 5 and Supplementary Table 4). No group difference was found between group connectivity maps after whole brain correction (*p* > 0.05, FWE).

### Trait, Region of Interest Blood Oxygenation-Level-Dependent Contrast, and Sex Effects on Connectivity

Psychological traits were not found to be associated with connectivity while controlling for NPY group after correction for comparisons of 12 trait measures (*p* > 0.05, linear model). Exploratory correlations between network measures and trait and state questionnaires are shown in Supplementary Figure 6. No differences were found between men and women in any of the static or dynamic connectivity measures according to standard two-sided *t*-tests (*p* > 0.05, two-sided *t*-test). The MID task salience BOLD contrast (high vs. low) for the nucleus accumbens, dorsal anterior insula, and dorsal anterior cingulate cortex were not correlated with any of the static or dynamic connectivity measures (*p* > 0.05, linear model).

## Discussion

NPY genotype has an impact on both static and dynamic functional connectivity in the salience network. Although no significant differences were found in functional connectivity maps from seeds in the NAc and dACC, in static network analyses of the salience network low-NPY subjects were found to have shorter path lengths, higher global efficiency, higher clustering, higher small-worldness, and higher node strength on average within the salience network. High-NPY subjects showed higher modularity and node eccentricity in the salience network. Salience network differences did not appear to be driven by any specific node, but rather by many nodes spread throughout the network. No differences were found between the groups in the default mode network. Dynamically, low-NPY subjects spent more time in a state displaying more coordination while high-NPY subjects spent more time in a state that had less coordinated connectivity in the salience network. High-NPY subjects also switched between states more often in this network. Again, no differences were found between groups in the default mode network.

We have previously shown that high and low-NPY subjects from the same sample differ in NAc activation during a monetary incentive delay task (Warthen et al., 2018). However, we did not find that functional connectivity maps based on the same NAc seed were statistically different between groups. These results indicate that, while NAc activation during reward behavior is different between groups, the two groups do not necessarily differ with respect to the information transfer to and from this region.

Because the salience network (Menon, 2015) and default mode network are implicated in many psychiatric disorders, we evaluated the impact of NPY expression on those networks. We found a hyperconnected salience network in low-NPY subjects. Similar findings have been observed in PTSD (Akiki et al., 2018; Fani et al., 2019) during rest as well as during eye-contact (Thome et al., 2014). In social and general anxiety, regions within the salience network (including the insula, anterior cingulate, and prefrontal cortex) have been observed to be hyperactive during rest and task functional connectivity (Etkin and Wager, 2007; Brühl et al., 2014). Structural hyperconnectivity between the anterior insula and amygdala, as measured with diffusion imaging, has also been shown to be associated with state and trait anxiety (Baur et al., 2013). Other studies on anxiety have reported heightened activity in the salience network (Sylvester et al., 2012), and point to increased error notification originating in the dACC (Hajcak et al., 2003; Paulus et al., 2004). Discordant results have also been observed, e.g., decreased connectivity in the salience network in anxiety (Geng et al., 2016; Xu et al., 2019). Increased coherence within the salience network may reflect increased vigilance in stimulus notification, which could lead to anxiety. Future work should examine the role of NPY in the activity of the salience network and how such network alterations impact risk for anxiety and depression.

Although low-NPY has been associated with depression, the hyperconnectivity within the salience network observed here has not been found in depression. A previous study showed a decrease in connectivity to the nucleus accumbens in subjects with depression compared to controls (Helm et al., 2018). Although we did not include this node in our salience network, we don’t see lower connectivity in low-NPY subjects in any node in the salience network when compared to high-NPY subjects. In ADHD, von Rhein et al. (2019) reported decreased functional connectivity between the salience network and executive control network, which may also point back to less coordinated functional connectivity within the salience network as we see in high-NPY subjects. Higher connectivity within the salience network may indicate a lower threshold for stimulus notification, which would fit symptoms seen in anxiety and PTSD. ADHD has been associated with lower functional connectivity within the salience network (von Rhein et al., 2019), as well as a possible higher necessary stimulus threshold for sustained attention (Tegelbeckers et al., 2015). The high-NPY group may share this parallel with subjects with ADHD.

The high-NPY group displayed a higher switching probability between states in the salience network. Subjects with ADHD have previously shown increased switching between networks (Cai et al., 2018; Scofield et al., 2019). Although here we examine within network switching probabilty, between and within network switching probabilities may be related. Depression has been shown to present with impaired salience network mediated switching into the central executive network (Wang et al., 2016), which may relate to the lower switching likelihood within the salience network shown here by the low-NPY group. Statically, we see lower long-distance global efficiency within the salience network in the high-NPY subjects, along with higher modularity. These findings are similar to network analyses in children with ADHD (Wang et al., 2009; Cai et al., 2018), which are of interest given that greater NPY function may be related to hyperactivity (Lesch et al., 2011), impulsivity (Bari et al., 2015), and possibly ADHD (Scassellati et al., 2012; Kourtesis et al., 2015).

The functional connectivity differences observed here between low and high-NPY groups seem to be specific to the salience network, as we did not find group differences in the default mode network. It would be reasonable to expect group differences in default mode network connectivity, as low-NPY is associated with the development of depression (Mickey et al., 2011), and stronger default mode network connectivity has been reported in subjects with depression (Wang et al., 2016; Helm et al., 2018), and low-NPY status has been associated with greater activation of medial prefrontal cortex (Mickey et al., 2011). The functional connectivity of those at risk for the development of depression may not look like the functional connectivity of those with active depression. PTSD, also associated with low-NPY (Yehuda et al., 2006), has been characterized by a generally hypoactive default mode network (Akiki et al., 2018), which we do not see in low-NPY subjects. This may indicate that a state change is necessary from the at-risk state to the disease state (e.g., depressed or PTSD) before differences in default mode network connectivity are observed. Overall these findings suggest that NPY exerts effects on risk for psychiatric disorders primarily through the salience network, and not the default mode network.

We found a negative relationship between salience network switching probability and global efficiency and clustering, but a positive relationship with characteristic path length. This is not surprising because a higher network switching rate should result in lower correlations between regions in the network over time, as they spend less time in coherent oscillations. A lower network switching rate would allow for stronger long-distance correlations, resulting in a higher characteristic path length in a weighted graph. Fractional occupancy showed these same relationships, a negative correlation with global efficiency and clustering but a positive association with characteristic pathlength, possibly for the same reason. Fractional occupancy also showed a positive correlation with modularity. A longer time spent in a certain network state, or higher fractional occupancy, may allow for stronger local correlations, resulting in higher modularity.

Our study has several limitations. Although the imaged sub-sample was genetically selected from a relatively large sample (>200), the number of imaged subjects was reduced, and many had to be excluded to avoid artifacts related to head motion. The resulting sample size limited the power to detect moderate-sized between-group effects. A replication sample would boost the strength of these findings, and this should be investigated further in the future but the practical limitations inhibit our ability to recruit another 200 subjects to sample from at this time. Furthermore, to address the multiple-comparison problem, we had to focus our hypotheses on two brain networks of interest. We recognize that there is no hard definition of what is and is not the salience or default mode network. These findings should be validated in alternate definitions of these networks in future studies. The distribution of NPY haplotypes in the general population also led to an imbalance of NPY group numbers which could impact the k-clustering algorithm. As a reviewer stated we do not know the circulating, CSF, or cellular levels of NPY in these subjects in the genetically predicted groups. As we are studying healthy humans, CSF and cellular levels of NPY are not feasible to obtain. Additionally, although we do not have circulating or serum levels of NPY these measures likely would not represent CSF levels of NPY (Dötsch et al., 1997). We suggest animal studies to determine the relationship of these measures of the brain regions of interest. Here we only examined resting-state functional connectivity; task-based functional connectivity analysis of the salience network may also provide additional insight into the effects of NPY on intrinsic functional networks. Finally, the resting state scans in this study were collected after a monetary incentive delay task for both groups. Previous studies have shown that prior tasks may impact resting state (Waites et al., 2005), however, the effect would be similar for both groups.

Generally, we found increased coordination across the salience network among low-NPY subjects, and a salience network that was less efficient and coherent among high-NPY subjects. Differences in connectivity in this network may point to differences in emotional regulation and salience signaling. These differences in processing may provide high-NPY subjects with resilience against depression, anxiety, and PTSD, but which also may put them at higher risk for attentional or hyperactive disorders. Stronger, more coordinated connectivity in the salience network may indicate a lower threshold for stimulus detection. In disorders of anxiety and PTSD, this may result in hyper-vigilance and constant notification of realistically irrelevant stimuli. Lower connectivity, as generally seen in cases of ADHD, may represent a higher necessary stimulus threshold for coherent activity of the salience network, or paying attention to the given stimulus. This lower connectivity may also result in higher switching probabilities within the network, generally representing less coordinated activity in the network over time. Anxiety, PTSD, and ADHD have all been suggested to stem from a lack of regulation in brain networks (Etkin and Wager, 2007; Hoekzema et al., 2014). Our results suggest that NPY may play a role in network regulation, and this should be a focus of future studies on neurobiological risk factors for anxiety and mood disorders.

## Conclusion

We have shown that low levels of NPY are associated with a more closely knit salience network, as determined by graph theory measures. Additionally, subjects with high levels of NPY displayed higher switching probabilities within the salience network, along with more time spent in a less strongly connected state. In the default mode network, no such differences were observed. NPY may exert effects on the risk of development of psychiatric disorders through subtly varied activity in the salience network.

## Data Availability Statement

The raw data supporting the conclusions of this article will be made available by the authors, without undue reservation.

## Ethics Statement

The studies involving human participants were reviewed and approved by the University of Michigan IRB. The patients/participants provided their written informed consent to participate in this study.

## Author Contributions

KW wrote the article, designed the analysis plan, and analyzed the data. RW developed some of the analytical tools, contributed to the analysis and analysis plan, and edited the article. BS collected the data. VK contributed to the analysis and analysis plan. MB contributed to the analysis plan and the initial data collection. BM edited the article, designed the initial data collection, collected the initial data, and contributed to the analysis and analysis plan. All authors contributed to the article and approved the submitted version.

## Conflict of Interest

The authors declare that the research was conducted in the absence of any commercial or financial relationships that could be construed as a potential conflict of interest.

## Publisher’s Note

All claims expressed in this article are solely those of the authors and do not necessarily represent those of their affiliated organizations, or those of the publisher, the editors and the reviewers. Any product that may be evaluated in this article, or claim that may be made by its manufacturer, is not guaranteed or endorsed by the publisher.

## Footnotes

1
https://github.com/juanitacabral/LEiDA
