# Supplementary material for: Neuropeptide Y Variation Is Associated With Altered Static and Dynamic Functional Connectivity of the Salience Network
Source: Front Syst Neurosci. 2021 Nov 18;15:629488. doi: 10.3389/fnsys.2021.629488 (PMC8636673; doi:10.3389/fnsys.2021.629488)
Supplement: Supplementary file 1 [file Data_Sheet_1.docx]

Supplementary Material

# Supplementary Introduction

Figure S1 shows visual examples of important graph theory concepts.


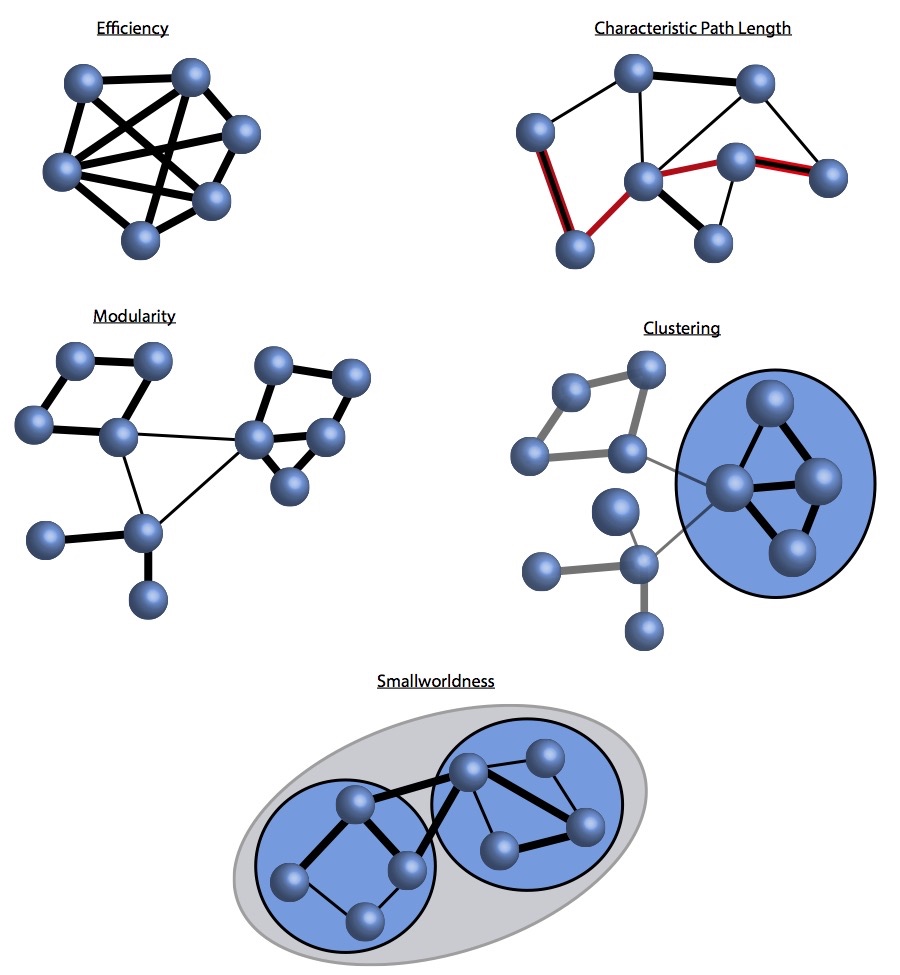


Figure S1., Examples of graph theory concepts

# Supplementary Results

## Salience Network Nodal Analysis

Higher node strength was found in the Low NPY group in the regions listed in table S1 and shown in figure S2 (A). Higher eccentricity was found in the High NPY group in the regions listed in table S2 and shown in figure S2 (B).

Table S1

Table S2

Figure S2. A. Nodes where strength is greater in Low NPY. B. Nodes where eccentricity is greater in High NPY.

## Default Mode Network Transitions

Figure S3 shows the transition rates of each group to and from each state in the default mode network. Group differences were found for the transition rate from state 1 to state 3 (p=0.024) and state 2 to state 3 (p=0.046), where the low NPY group was more likely to switch into state 3 in both cases. These values did not survive Holm’s test for multiple comparisons (p>0.05).

Figure S3. Switching rates between states in the default mode network for low (top values) and high (bottom values) NPY groups. *p<0.05 as determined by a standard two-sided t-test.

## Seed-based functional connectivity

Figure S4. NPY group comparison of NAc functional connectivity and dAcc functional connectivity. A. NAc seed, Low-NPY connectivity > High-NPY (-23, -42, -16). B. NAc seed, High-NPY connectivity > Low-NPY (6, 32, -11). C. dAcc seed, Low-NPY connectivity > High-NPY (-44, 18, -9). D. dAcc seed, High-NPY > Low-NPY (14, -54, 16). No voxels survived whole brain correction for any contrast. Display threshold: p<0.01 uncorrected.


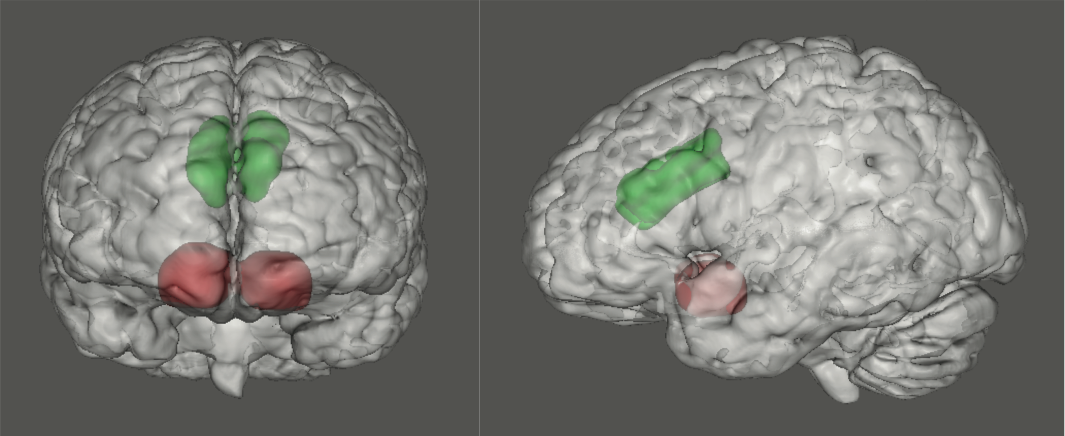


Figure S5. dAcc (green) and NAc (red) regions of interest used for seed-base functional connectivity

Table S3. Seed-based connectivity of the NAc and dACC.

| A. Salience Network  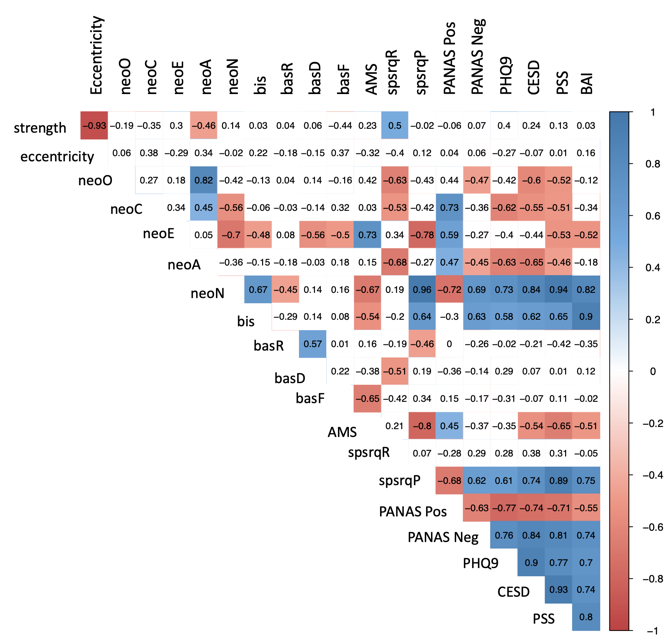 | B. Default Mode Network  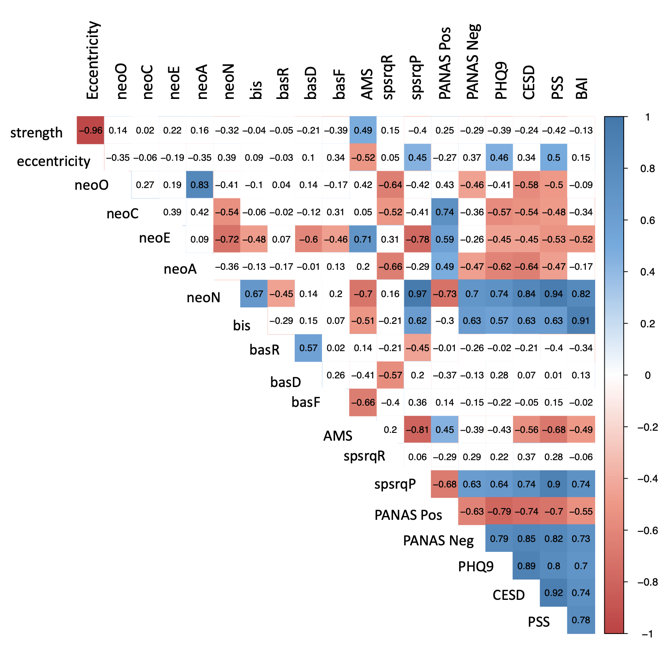 |
| --- | --- |
| Figure S6. Correlations of Stationary Network Measures with States and Traits. A. Correlations between strength and eccentricity of the salience network and state and trait measures. B. Correlations between strength and eccentricity of the default mode network and state and trait measures. neoO: Revised NEO Personality Inventory Openness, neoC: NEO Conscientiousness, neoA: NEO Agreeableness, neoN: NEO Neuroticism, bis: BIS/BAS behavioral inhibition scale, basR: BIS/BAS reward scale, basD: BIS/BAS drive scale, basF: BIS/BAS fun seeking scale, AMS: Automated Neuropsychological Assessment Metrics Mood Scale, spsrqR: Sensitivity to Punishment and Sensitivity to Reward Questionnaire, reward, spsrqP: Sensitivity to Punishment and Sensitivity to Reward Questionnaire, punishment, PANAS Pos: Positive and Negative Affect Scale, positive, PANAS Neg: Positive and Negative Affect Scale, negative, PHQ9: depression Patient Health Questionnaire, CESD: Center for Epidemiologic Studies Depression Scale, PSS: Perceived Stress Scale. Correlations are shown in their respective color (blue for positive correlations, red for negative correlations) if p<0.05 as determined by cor.mtest in R, uncorrected. | |

In an exploratory analysis we evaluated the correlations between network measures of strength and eccentricity and state and trait measures. We only included strength and eccentricity given the highly correlated or anti-correlated nature of the network data. Higher strength in the salience network was inversely correlated with neo agreeableness and correlated with spsrq reward. Strength of the default mode network was correlated with the AMS scale, while increased eccentricity of the default mode network was inversely correlated with the AMS and positively correlated with the spsrqP, the PHQ9, and the PSS.

## Quality Control

DVARS was calculated according to Afyouni et al. (2018), values for each included subject are shown below in Table S4.

Table S4.

Figure S7. NPY group movement as shown by mean frame displacement (mm). Motion cutoffs shown with the blue line at 0.2 mm.

# Citations

Afyouni, S., and Nichols, T. E. (2018). Insight and inference for DVARS. *Neuroimage* 172, 291–312. doi:10.1016/j.neuroimage.2017.12.098.
